# Laminin-Coated Electrospun Regenerated Silk Fibroin Mats Promote Neural Progenitor Cell Proliferation, Differentiation, and Survival *in vitro*

**DOI:** 10.3389/fbioe.2019.00190

**Published:** 2019-08-06

**Authors:** Guangfei Li, Kai Chen, Dan You, Mingyu Xia, Wen Li, Suna Fan, Renjie Chai, Yaopeng Zhang, Huawei Li, Shan Sun

**Affiliations:** ^1^NHC Key Laboratory of Hearing Medicine, State Key Laboratory of Medical Neurobiology, Shanghai Engineering Research Centre of Cochlear Implant, Otorhinolaryngology Department of Affiliated Eye and ENT Hospital, Ear, Nose & Throat Institute, Fudan University, Shanghai, China; ^2^State Key Laboratory for Modification of Chemical Fibers and Polymer Materials, International Joint Laboratory for Advanced Fiber and Low-Dimension Materials, College of Materials Science and Engineering, Donghua University, Shanghai, China; ^3^Key Laboratory for Developmental Genes and Human Disease, Ministry of Education, Jiangsu Province High-Tech Key Laboratory for Bio-Medical Research, Institute of Life Sciences, Southeast University, Nanjing, China; ^4^Collaborative Innovation Center for Brain Science, Institute of Biomedical Sciences, Institute of Brain Science, Fudan University, Shanghai, China

**Keywords:** regenerated silk fibroin mats, neural progenitor cells, biocompatibility, proliferation, differentiation

## Abstract

Neural progenitor cell (NPC) transplantation is a promising technique for central nervous system (CNS) reconstruction and regeneration. Biomaterial scaffolds, frameworks, and platforms can support NPC proliferation and differentiation *in vitro* as well as serve as a temporary extracellular matrix after transplantation. However, further applications of biomaterials require improved biological attributes. Silk fibroin (SF), which is produced by *Bombyx mori*, is a widely used and studied protein polymer for biomaterial application. Here, we prepared aligned and random eletrospun regenerated SF (RSF) scaffolds, and evaluated their impact on the growth of NPCs. First, we isolated NPCs and then cultured them on either laminin-coated RSF mats or conventional laminin-coated coverslips for cell assays. We found that aligned and random RSF led to increases in NPC proliferation of 143.8 ± 13.3% and 156.3 ± 14.7%, respectively, compared to controls. Next, we investigated neuron differentiation and found that the aligned and the random RSF led to increases in increase in neuron differentiation of about 93.2 ± 6.4%, and 3167.1 ± 4.8%, respectively, compared to controls. Furthermore, we measured the survival of NPCs and found that RSF promoted NPC survival, and found there was no difference among those three groups. Finally, signaling pathways in cells cultured on RSF mats were studied for their contributions in neural cell differentiation. Our results indicate that RSF mats provide a functional microenvironment and represent a useful scaffold for the development of new strategies in neural engineering research.

## Introduction

Numerous tissue engineering methods have been developed as a means to replace damaged or diseased organs (Langer and Vacanti, 2016). The essential factors in nerve tissue engineering include the cells that are used, growth factors like insulin-like growth factor, and bone morphogenetic proteins (BMPs) (Nyberg et al., 2016; Syverud et al., 2016), and the materials and implantation methods. In the case of the same implantation method, the choice of cells and biological materials is particularly important for nerve regeneration. These approaches use tissue-specific cells that are grown on a scaffold material with the purpose of creating a functional tissue or organ. Many biomaterials are used in tissue engineering, including both synthetic and natural materials (Unger, 2004) such as graphene film (He et al., 2016), polycaprolactone (Saadatkish et al., 2018), poly(lactic-coglycolic acid) (Hualin, 2011), poly(L-lactide-cocaprolactone) (Su et al., 2012), silk fibroin (SF) (Xie et al., 2014), starch (Salgado et al., 2004), collagen (Bozkurt et al., 2017), gelatin (Su and Wang, 2015), or chitosan (Vårum et al., 1997).

The central nervous system (CNS) and peripheral nervous system have limited capacity for regeneration after traumatic injury or disease, resulting in functional paralysis of the nervous system. Therefore, effective methods of nerve repair and regeneration for functional recovery are urgently needed. In recent years, considerable attention has been focused on tissue engineering with nanofibers, including functionalized scaffolds. However, there remain many challenges before full functional recovery of the nervous system will be possible, especially in terms of regenerating non-renewable neurons. Neural progenitor cells (NPCs) are self-renewable and multipotent stem cells, i.e., they can make copies of themselves and can differentiate into many different mature cell types (Gage and Temple, 2013), and much work has focused on growing NPCs on tissue-engineered scaffolds as part of cell-based transplantation therapies for treating a variety of CNS diseases and for repairing nerve injuries. At its most basic, an effective scaffold should provide a suitable substrate for specific types of cells to grow on. In addition, a main function of a tissue-engineered scaffold is to guide cell behaviors, such as growth and survival, via cell—matrix and cell—cell interactions that facilitate sensing and responding to the environment.

However, many biomaterials are not highly biocompatible with host tissues and might trigger inflammatory and immune reactions or foreign body reactions after implantation. For example, some synthetic biomaterials such as hybrid bio-glass induce a mild level of inflammation (Ravarian et al., 2013), and many studies have suggested that synthetic bone substitute materials with different compositions can trigger inflammatory reactions (Al-Maawi et al., 2017). In addition, induction of the foreign body reaction can inhibit or prevent cell regeneration and migration and integration to the host.

SF, which is produced from the silkworm, *Bombyx mori*, has been used as a promising biomaterial for centuries due to its excellent biocompatibility and biodegradability *in vitro* and *in vivo* as well as its outstanding mechanical strength (Altman et al., 2003; Meinel et al., 2005), its low level of inflammatory response (Meinel et al., 2005), and its versatility in processing (Wei et al., 2011). It has been used as suture material for surgical operations for a very long time (Choudhury et al., 2016), and it has been successfully processed into different forms, including but not limited to films (Jin et al., 2005), 3D porous scaffolds (Kim et al., 2005), hydrogels (Park et al., 2014), sponges (Silva et al., 2008), and fibers via wet spinning (Yan et al., 2010), or electrospinning (He et al., 2011). Among these forms, electrospun regenerated silk fibroin (RSF) has many advantages such as high specific surface area, appropriate porosity, and nanoscale diameter (Deitzel et al., 2001; Ma et al., 2005; Thomas et al., 2006). The high specific surface area of electrospun RSF mats can provide more surface area for cell growth, and the RSF nanofibers making up the mats facilitate cell adhesion. The pore size and porosity of RSF mats are beneficial to cell growth (Ki et al., 2008) in terms of the formation of extracellular matrix, the transport of oxygen and nutrients, and the removal of metabolites (Smith and Ma, 2004). RSF has been used as scaffold material to support cell adhesion, proliferation, and differentiation *in vitro* and as tissue scaffolds *in vivo* to promote tissue repair such as cartilage (Silva et al., 2008; Bhardwaj et al., 2011), bone (Mauney et al., 2007), blood vessels (Fukayama et al., 2015), and urinary vessels (Xie et al., 2014); to promote wound healing (Liu et al., 2010); and to deliver drugs (Yucel et al., 2014; Zhao et al., 2015). However, it is not fully understood yet whether NPC growth, proliferation, or differentiation can be improved using electrospun RSF biomaterials. This is important for the design of a suitable advanced tissue engineering biomaterial for the nervous system. Increasing evidence suggests that the biopolymers should present as porous 3D structures that mimic the size and scale of the fibers composing the extracellular matrix (ECM) of native nerve tissue and organs (Crapo et al., 2011; Jang et al., 2017). Electrospinning is an common nanotechnology approach that allows for the consistent production of fibers with a specific diameter and thus has been applied in studies of NPC proliferation and differentiation and subsequent nerve regeneration at the injured site (Bai et al., 2014; Dinis et al., 2015; Boni et al., 2018; Bhattarai et al., 2019).

Cellular signaling pathways play vital roles in NPC proliferation and differentiation, including the Sonic hedgehog (SHH) signaling pathway and BMP4 signaling pathway. In some contexts during development, Shh regulates neural progenitor cell differentiation, survival, and proliferation (Ruiz i Altaba et al., 2002), and it regulates midbrain and forebrain dopaminergic and serotonergic neuronal differentiation (Hynes et al., 1995). In other contexts, however, Shh promotes differentiation into oligodendrocytes and astroglia (Dahmane and Ruiz i Altaba, 1999). The BMP4 signaling pathway is a key player in regulating neuronal and glial cell development from neural progenitor cells in the embryonic, postnatal, and injured CNS (Cole et al., 2016). BMP4 has a significant and temporally dependent influence on both neuronal and glial differentiation of NPCs (Hegarty et al., 2013), and BMP4 also promotes astrocytic differentiation through multiple mechanisms (Gross et al., 1996).

In this study, we investigated the attachment, viability, growth, proliferation, and differentiation capacities of NPCs from the embryonic mouse hippocampus on both laminin-coated aligned and random RSF mats, which were prepared free of sericin, which is known to trigger inflammatory reactions (Aramwit et al., 2009). Furthermore, the RSF mats can be functionalized by the covalent attachment of cell adhesion molecules and can influence cellular signaling pathways, thus we investigated the signaling pathways that might be involved in NPC differentiation. In addition, the difference between aligned and random RSF was also investigated to determine whether the surface characteristics or the structure of synthetic RSF are involved in cell fate determination. Our objective was to determine the suitability of RSF mats as biomaterial templates for NPC culture.

## Materials and Methods

### Preparation of RSF Mats

Cocoons of *B. mori* were purchased from Tongxiang, China. Semipermeable cellulose membranes with a molecular weight cutoff of 14,000 ± 2,000 Da were purchased from Yuanju Co., Ltd. (Shanghai, China). All other chemicals were of analytical grade and were purchased from Sinopharm Chemical Reagent Co. Ltd. (Shanghai, China).

In order to remove the sericin, the cocoons were degummed in 0.5 wt% Na_2_CO_3_ aqueous solution at 100°C for 30 min and rinsed repeatedly with deionized water. The degummed silk fibers were then dried at 4°C overnight and immersed the next day in 9.0 M LiBr aqueous solution at 40°C for 2 h to completely dissolve them. The solution was centrifuged at 1,200 × g for 10 min at 10°C to remove undissolved impurities. Next, the supernatant was placed in a semipermeable cellulose membrane and dialyzed against deionized water for 3 days to remove LiBr ions. The obtained RSF solution was concentrated to 33 wt% by forced air flow for further experiments. According to the non-gel sieving capillary electrophoresis (NGSCE) results, it was found that the RSF had an molecular weight (MW) around 83 kDa with a wide MW distribution (MWD). The RSF molecules are mainly the segments of the heavy chain of natural B. mori silk fibroin (Wei et al., 2010).

The traditional method to electrospinning RSF mats was performed as described by Li (2015), while the preparation of electrospun RSF mats with aligned fibers was performed as described by Liu (2016). For both methods, a syringe containing 33 wt% RSF dope was run through a stainless-steel needle with an inner diameter of 0.6 mm using a syringe pump with a flow rate of 1.2 mL/h. A voltage of 20 kV was applied between the spinneret and collector, with a distance of 12 cm. The spinning temperature was kept at room temperature, and the relative humidity was 50 ± 5%. For conventional electrospun RSF mats with random fibers, the collector was an aluminum foil-coated steel plate placed perpendicular to the needle. For electrospun RSF mats with aligned fibers, the collector was an aluminum foil-coated roller rotating at a speed of 2,000 rpm. The as-spun RSF mats were peeled off the collectors and placed in a test chamber at a constant 90% relative humidity and 37°C for 36 h to increase the crystallinity of the mats, and the thickness of the samples were measured by a thickness gauge. Before cell seeding, the post-treated RSF mats were soaked in 75 vol% ethanol for 2 h, then washed with sterilized PBS and cell culture medium three times and coated with laminin solution (5 mg/mL, Sigma-Aldrich) in PBS for at least 4 h at 37°C prior to soaking in proliferation medium overnight.

### Mechanical Properties

Tensile properties of the post-treated RSF mats (35 × 5 mm) were measured using an Instron 5,969 material testing machine at 20 ± 5°C and 50 ± 5% relative humidity. Samples were tested at an extension rate of 3 mm/min at a gauge length of 20 mm. The thickness of each mat sample was measured 10 times by a CH-1-S thickness gauge (Shanghai Liuling Instruments Co., Shanghai, China) with a resolution of 1 μm (Li, 2015). In the case of the RSF mats with aligned fibers, the tensile strength was measured along the direction of the fiber alignment.

### Isolation and Culture of the NPCs

Two different media were used in this study. The proliferation medium consisted of 2% B27 (Life Technologies), 20 ng/mL epidermal growth factor (Thermo Fisher Scientific, Rockford, IL, USA), 10 ng/mL basic fibroblast growth factor (Invitrogen), and 1% penicillin-streptomycin (BBI Life Sciences) in DMEM/F12 (Gibco). The differentiation medium consisted of 2% B27, 1% fetal bovine serum (Life Technologies), 1 μM retinoic acid (Sigma-Aldrich, USA), and 1% penicillin-streptomycin in DMEM/F12.

As previously described (Guo et al., 2016), the NPCs were extracted and purified from the hippocampus of embryonic day 16 ICR mice (adult mice for producing embryos were purchased from Shanghai Jiesijie Animal Experiment Co., Ltd.) and then seeded at a concentration of ~5 × 10^4^ cells/mL in a 25 cm^3^ culture flask (Corning) in proliferation medium to form NPC neurospheres. For proliferation studies, the NPCs were seeded at a concentration of ~8 × 10^4^ cells/mL in proliferation medium. For differentiation studies, the NPCs were seeded at a concentration of ~8 × 10^4^ cells/mL in differentiation medium. For cell seeding, the neurospheres were gathered and enzymatically digested with Accutase (Life Technologies) to obtain a suspension of single cells. After seeding, the RSF mats together with seeded NPCs were moved to 24-well cell culture plates (Corning). Cells in 25 cm^3^ culture flask were routinely passaged 1:2, and for propagation, the NPC neurospheres were collected after 7 days in culture and dissociated with Accutase at 37°C for 7 min followed by the addition of 0.01 M PBS to block the reaction. The cells were then re-plated into 25 cm^3^ culture flasks, and the propagation was repeated at 7-day intervals. All cells were used at low passage numbers (*N* = 5–10). All animal procedures were performed according to protocols approved by the Animal Care and Use Committee of Fudan University and were consistent with the National Institutes of Health Guide for the Care and Use of Laboratory Animals. All efforts were made to minimize the number of animals used and to prevent their suffering.

### Field Emission Scanning Electron Microscope Observation of NPCs Cultured on RSF Mats

For imaging the morphology of NPCs on different substrates, cells were fixed in 4% paraformaldehyde for 2 h and then dehydrated with a series of graded ethanol solutions [30, 50, 70, 75, 80, 90, and 100% (v/v)]. The ethanol was removed and tert-butanol was added. Finally, the SF mats were frozen at −50°C for 4 h and then freeze-dried overnight. After sputter coating with platinum, the surface morphology of the prepared RSF mats and the prepared NPCs on RSF mats was imaged on a field emission scanning electron microscope (SEM) (SU8010, Hitachi, Japan) operating at an accelerating voltage of 10 kV. One hundred fibers in the SEM image were randomly selected, and the average diameter of the fibers was measured with the ImageJ software.

### Cell Viability Assay

After 5 days of culture in proliferation medium, a cell viability test was performed using ethidium homodimer (Abcam) and calcein-AM (Abcam) according to the manufacturer's instructions. First, the NPCs cultured on the laminin-coated coverslip and on the RSF mats were rinsed three times in sterile 0.01 M PBS. Then the cells were incubated in sterile 0.01 M PBS containing 2 × 10^−4^ mM of calcein-AM and 2 × 10^−4^ mM ethidium homodimer for 30 min at 37°C. After incubation, the cells were rinsed three times in 0.01 M PBS for 10 min. The live NPCs labeled with calcein-AM showed green color and the dead NPCs stained with ethidium homodimer showed red color under a Leica SP8 confocal microscope. The percentage of living cells was calculated by dividing the number of calcein-AM–positive cells by the total cell number.

### Cell Counting Kit-8 (CCK-8) Assay

The NPCs were cultured for 1, 3, 5, and 7 days in proliferation medium and then used with the CCK-8 cell proliferation assay and cytotoxicity assay kit (DOJINDO, Japan) according to the manufacturer's instructions. In brief, NPCs were transferred to 24-well plates with coverslips or with aligned or random RSF mats and maintained in a humidified atmosphere with 5% CO_2_ at 37°C. Then 10% v/v CCK-8 solution was added to the culture medium at the indicated time points and the wells were incubated for 4 h. Finally, 100 μL of cell solution was transferred to a 96-well plate and the absorbance at 450 nm was measured using a microplate reader.

### Immunofluorescence

After 5 days of culture in proliferation medium and 7 days of culture in differentiation medium, NPCs cultured on RSF mats and laminin-coated coverslips were washed once with 0.01 M PBS, fixed in ice cold 4% paraformaldehyde in 0.01 M PBS for 30 min, washed with 0.01 M PBS three times for 10 min, permeabilized with 1% Triton-X100 in 0.01 M PBS (1% PBS-T) for 30 min, and blocked with 10% BSA in 0.01 M PBS for 1 h at 37°C. The samples were incubated with the primary antibody overnight at 4°C and then incubated at 37°C for 1 h. The next day unbound primary antibodies were rinsed with 0.01 M PBS three times for 10 min and secondary antibodies were incubated for 1 h at room temperature. Unbound antibodies were then rinsed with 0.01 M PBS three times for 10 min followed by DAPI staining for 15 min. The Click-iT EdU Imaging Kit (Life Technologies) was used to detect cell proliferation according to the manufacturer's instructions. The primary antibodies used in this study included mouse anti-nestin IgG1 (1:1,000 dilution, Abcam), rabbit anti-neuron-specific class III beta-tubulin (Tuj-1, 1:1,000 dilution, Abcam), mouse anti-glial fibrillary acidic protein (GFAP, 1:1,000 dilution, Sigma-Aldrich), rabbit anti-Ki-67 (1:1,000 dilution, Abcam), and rabbit anti-vinculin (1:500 dilution, Abcam). The corresponding secondary antibodies were cy3 and 488 (1:500 dilution, Jackson ImmunoResearch). The nuclei were stained with DAPI (1:800 dilution, Sigma-Aldrich). All antibodies used in this study were diluted in 1% PBS-T.

### Terminal Deoxynucleotidyl Transferase dUNT Nick End Labeling (TUNEL) Detection

The potential cytotoxicity of the RSF mats was measured by detecting apoptosis-related DNA fragmentation with a TUNEL staining kit (Click-iT® Plus TUNEL Assay for *in situ* Apoptosis Detection, Invitrogen) according to the manufacturer's instruction. The nuclei were labeled with DAPI (1:800 dilution), and the cells were evaluated by confocal microscopy (Leica, SP8, Germany).

### Western Blotting

Cells were harvested with Accutase and lysed in RIPA and PMSF buffer (99:1) on ice for 30 min to 1 h. Total protein was isolated and the concentration was measured using a bicinchoninic acid kit (Thermo Fisher Scientific). The collected protein samples were loaded on a 12% sodium dodecyl sulfate-polyacrylamide gel, separated by gel electrophoresis, and transferred to polyvinylidene difluoride membranes (Immobilon-P; Millipore, Bedford, MA, USA). The membranes were blocked in 5% nonfat dried milk in 50 mM Tris-HCl (pH 7.4), 150 mM NaCl, and 0.1% Tween-20 (TBST) at room temperature for 1 h and then incubated with primary antibodies at 4°C overnight. The primary antibodies were anti-nestin (1:1,000 dilution, Abcam), anti-Tuj-1 (1:1,000 dilution, Abcam), anti-GFAP (1:1,000, dilution, Sigma-Aldrich), anti-Ki-67 (1:1,000 dilution, Abcam), and anti-GAPDH (1:1,000 dilution). Unbound primary antibodies were washed away with TBST three times for 10 min, and the membranes were incubated with goat anti-rabbit (1:1,000 dilution) and goat anti-mouse (1:1,000 dilution) secondary antibodies at room temperature for 1 h and then washed with TBST three times for 10 min. Finally, the ECL Detection Reagent (Pierce, USA) was used to measure protein expression with GAPDH as the internal control.

### RNA Extraction and RT-PCR

The NPCs were cultured in differentiation medium for 7 days, then the total RNA was extracted from the NPCs cultured on the coverslips and RSF mats using TRIzol (Invitrogen, Carlsbad, CA, USA). This was followed by complementary DNA synthesis using the GoScript Reverse Transcription System (Promega, Madison, WI, USA). qPCR reactions were performed with GoTaq® qPCR Master Mix (Promega) on a 7500HT Fast Real-Time PCR System (Applied Biosystems, Foster City, CA, USA). Each PCR reaction was carried out in triplicate. The mRNA levels were normalized to GAPDH (internal control), and the relative quantification of gene expression was analyzed using the ΔΔCT method (Livak and Schmittgen, 2001). The primer sequences are shown in Table 1.

**Table 1:** Sequences of PCR primers.

**Primer**	**Forward (5^**′**^**→** 3^**′**^)**	**Reverse (5^**′**^**→** 3^**′**^)**
Nestin	TTGCCTAATACCCTTGAGACT	TGGGAGGACACCAGTAGAAC
Tuj-1	CTGTTCAAACGCATCTCG	TCATCATCTTCATACATCTCCC
GFAP	AAGGAGCCCACCAAACTG	GCAAACTTAGACCGATACCAC
Olig2	TCACCTCCGACGCCAAGT	TTCAGCCAAAGAGTCAACCAG
Smo	TGTGAGAATGACCGAGTGGA	GCAGGGTAGCGATTGGAG
Gli 1	CCAAGCCAACTTTATGTCAGGG	AGCCCGCTTCTTTGTTAATTTGA
Gli 2	CAACGCCTACTCTCCCAGAC	GAGCCTTGATGTACTGTACCAC
Gli 3	CACAGCTCTACGGCGACTG	CTGCATAGTGATTGCGTTTCTTC
BMP 4	ACTGCCGTCGCCATTCAC	CACCACCTTGTCATACTCATCC
Id 1	CAAACCGCAGACCAAGAA	TCACCAAAGCGTCCACAG
Id 2	AAAGCACCTTGTGGAATC	TAACGGAGAAGTGGGAAT
Id 3	AAGAAGGGTGCTATGGAG	ATACTGGAGGTAAGACTGG

### Statistical Analysis and Cell Counting

For every experiment in all conditions studied, six random images obtained at 40 × magnification with the Leica SP8 laser confocal microscope were picked for manual quantification. Judged by DAPI staining, each such micrograph depicted around 150 to 1,000 cells in fields with a diameter of around 500 mm. Microsoft Excel and GraphPad Prism 6.0 (GraphPad Software, San Diego, California) were used for statistical analysis of the data. Unpaired Student's *t*-tests were used to determine statistical significance when comparing two groups, and one-way ANOVA followed by a Dunnett's multiple comparisons test was used when comparing more than two groups. *P*-values < 0.05 were considered statistically significant. Data are shown as the mean ± SEM.

## Results

### Preparation and Characterization of RSF Mats

**Figure 1:**
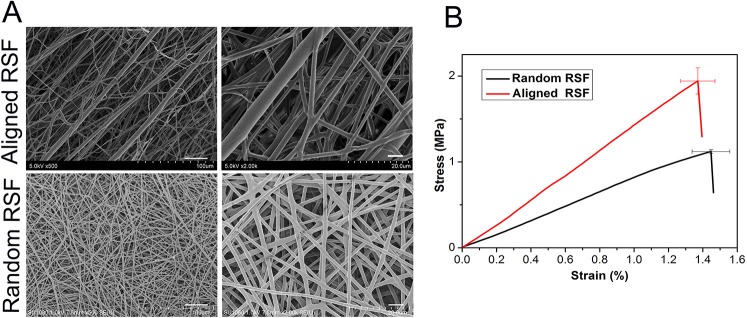
SEM images **(A)** and tensile strength **(B)** of electrospun RSF mats with aligned fibers and randomly collected fibers. Scale bar: 50 μm.

The fiber surfaces of the aligned and random RSF mats were smooth and uniform in diameter. The average thickness of these two types of RSF mats is about 0.12 mm. The fibers of the conventional electrospun RSF scaffolds were randomly distributed, while the fibers of the modified electrospun RSF mats were aligned along the rotating direction of the collector. The reason for this alignment was that the Taylor cone of the RSF dope was subjected to a certain traction force from the high-speed rotation of the roller (Figure 1A). The breaking strength of the RSF mats with aligned fibers were improved from 1.12 ± 0.02 MPa to 1.94 ± 0.15 MPa (Figure 1B).

### NPC Adhesion on RSF Mats

**Figure 2:**
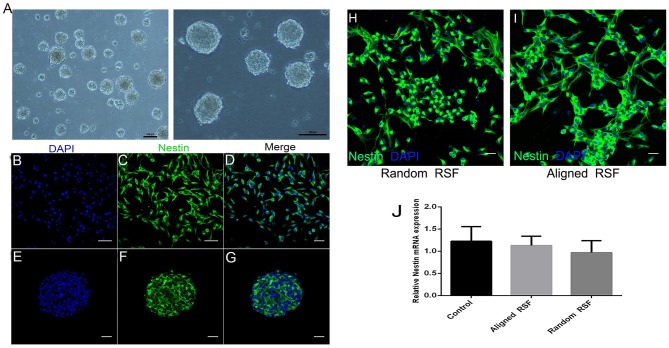
Characterization of NPCs. **(A)** Neurosphere formation of NPCs at DIV 5. Immunostaining against nestin (green), an NPC marker, in the individual NPCs **(B–D)** and neurospheres **(E–G)**. Immunostaining against nestin (green) with NPCs on random and aligned RSF mats **(H,I)**. mRNA expression of Nestin in NPCs grown on aligned and random RSF mats and laminin-coating coverslips for 5 days **(J)**. Nuclei were stained with DAPI (blue). Scale bar: 50 μm.

**Figure 3:**
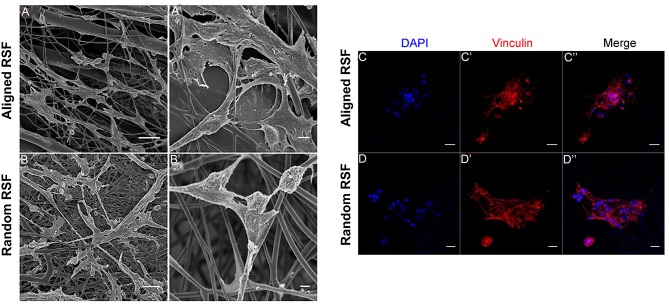
NPC adhesion on random and aligned RSF mats after 3 days of culture in proliferation medium. Low-magnification **(A,B)** and high-magnification **(A,B)** SEM images of NPCs cultured on laminin-coated random and aligned RSF mats in proliferation medium. The high-magnification SEM images illustrate the interaction between the cell filopodia and the RSF mat surface. Immunostaining against vinculin in NPC progeny cultured on the aligned **(C–C”)** and random RSF mats **(D–D”)** in proliferation medium after 3 days culture. Scale bar: 50 μm.

As in previous literature, several methods were used to detect NPCs. After 5 days of culture under proliferation conditions, we observed the formation of free-floating neurospheres (Figure 2A), which indicated the presence of NPCs. Also, immunostaining against nestin, an NPC marker, showed that most of the cells were nestin positive (Figures 2B–G), further validating the NPCs in this study. After 3 days of culture under proliferation and differentiation conditions, the characteristics of the NPCs were carefully examined on the RSF mats. Because cell adhesion plays a vital role in regulating cell growth, migration, differentiation, proliferation, and apoptosis, we investigated the adhesion of NPCs on RSF mats. After culturing for 3 days, the NPCs seeded on the aligned or random RSF mats adhered to the surface of the mats (Figures 3A,B). SEM images showed that the NPCs extended pseudopodia and established connections with each other after 3 days of culture in proliferative medium on aligned and random RSF mats (Figures 3A,B). In addition, we observed cell adhesion on RSF mats without laminin coating according to SEM (Figure S1). Vinculin, a cytoskeleton protein, plays an important role in cell adhesion, stretching, movement, proliferation, and survival activities by combining and interacting with a variety of cytoskeleton proteins and cytoskeleton f-actin to participate in cell chemical signal transduction. Immunostaining for vinculin after 3 days of culture showed that NPCs could adhere to the RSF mats along the RSF fibers (Figures 3C–C”,D–D”).

### Biocompatibility of the RSF Mats

**Figure 4:**
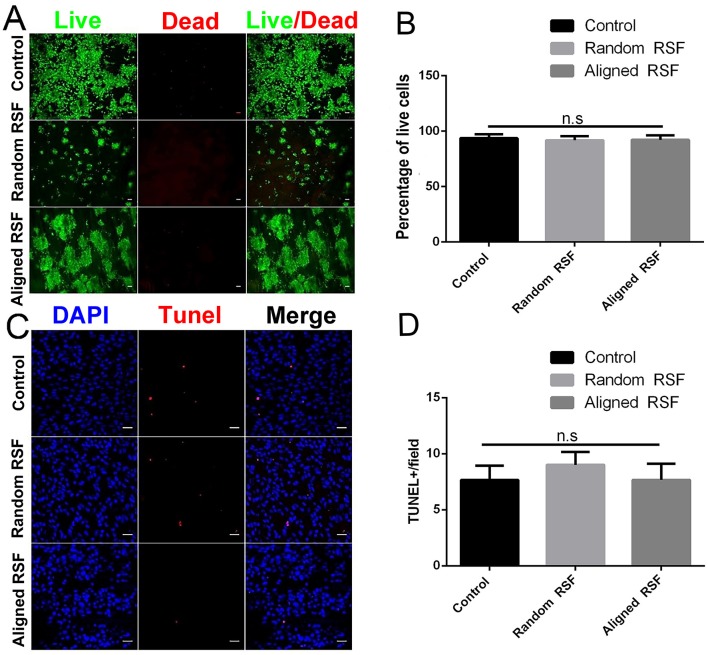
**(A)** Cell viability assay of NPCs on random and aligned RSF mats after 5 days of culture as determined by live/dead assay. Live cells are stained green and dead cells are red. Scale bar: 50 μm. **(B)** The percentage of live cells on the coverslip and the random and aligned RSF mats. **(C)** Representative photographs of TUNEL staining (which detects DNA fragmentation as an indicator of apoptosis) in NPCs cultured on coverslips and random and aligned RSF mats. All nuclei were counterstained with DAPI in blue, and all TUNEL-positive cells are red. Scale bar: 20 μm. **(D)** The average number of TUNEL-positive nuclei per field is shown (*n* = 20 fields for all three substrates).

The cytotoxicity of the aligned and random RSF mats was evaluated by calcein-AM and ethidium homodimer staining assay with laminin-coated coverslips as the control. After 5 days of culture, almost 95% of the attached cells were viable on all three substrates (Figures 4A,B), and the TUNEL assay showed no significant differences in apoptosis for any of the substrates (Figures 4C,D). This indicates that RSF mats have good biocompatibility, which is consistent with previous studies (Jiang et al., 2014). Cells cultured on RSF mats were also stained with antibodies against nestin. Nearly all of cells on the aligned and random RSF mats were immunopositive for nestin after 5 days of culture (Figures 2H,I), with no obvious difference compared to the laminin-coated coverslip. Furthermore, the real-time PCR analysis showed similar results for Nestin expression in NPCs grown for 5 days on aligned and random RSF mats and laminin-coating coverslips (Figure 2J), suggesting that NPCs grew well on the aligned and random RSF mats and maintained their stemness.

### Proliferation of NPCs Cultured on Aligned and Random RSF Mats

**Figure 5:**
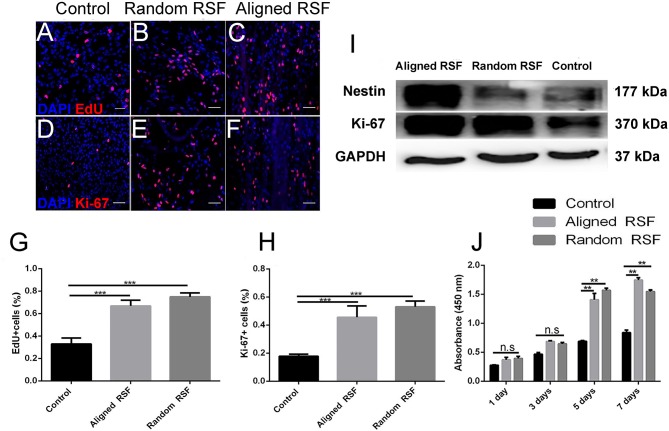
NPC proliferation on coverslip controls and random and aligned RSF mats after culturing for 7 days. The expression of EdU on control **(A)**, random RSF mats **(B)**, and aligned RSF mats **(C)** and the respective expression of Ki-67 **(D–F)**. The percentages of EdU+ cells **(G)** and Ki-67+ cells **(H)** in the three groups. **(I)** Western blot analysis of Ki-67 and nestin expression on coverslip controls and random and aligned RSF mats. **(J)** The histogram depicts the CCK-8 assay on the coverslip controls and RSF mats after culturing for 1, 3, 5, 7 days in proliferation medium, respectively. The data are presented as mean ± standard error of the mean, ^**^p < 0.01, ^***^p < 0.001. Scale bar: 50 μm.

NPC proliferation on the aligned and random RSF mats was assayed by immunostaining for Ki-67, which is a nucleoprotein involved in the cell cycle and is an indicator of cell proliferation activity. As we expected, after 7 days of culturing in proliferation medium 19.49 ± 1.07% (SE) of the cells on the laminin-coated coverslip were Ki-67 positive (Figure 5D), while almost 43.85 ± 0.9% and 40.6 ± 1.06% of the NPCs were Ki-67 positive on the random (Figure 5E) and aligned RSF mats (Figure 5F), respectively. Thus, cell proliferation was significantly improved on RSF mats compared to laminin-coated coverslips (Figure 5H). EdU, which is a thymine nucleoside analog, can be incorporated in place of thymine during the DNA synthesis phase of the cell cycle and thus also acts as a marker of proliferation. The percentages of EdU-positive cells grown on random (Figure 5B) and aligned RSF mats (Figure 5C) were 60.47 ± 1.1% and 57.66 ± 1.27%, respectively, compared to 24.23 ± 1.1% on the coverslip (Figure 5A). Thus, the percentages of Ki-67–positive cells were analogous to the percentages of EdU-positive cells (Figures 5G,H). Western blot analysis demonstrated that the protein expression of Ki-67 in NPCs cultured on the aligned and random RSF mats was significantly greater than on the coverslips (Figure 5I). This suggests that NPCs on the RSF mats sustained a more active NPC proliferation state compared to the coverslips (consistent with the CCK-8 cell proliferation assay after culturing for 1, 3, 5, 7 days in proliferation medium respectively, Figure 5J).

### Differentiation of NPCs on Aligned and Random RSF Mats

**Figure 6:**
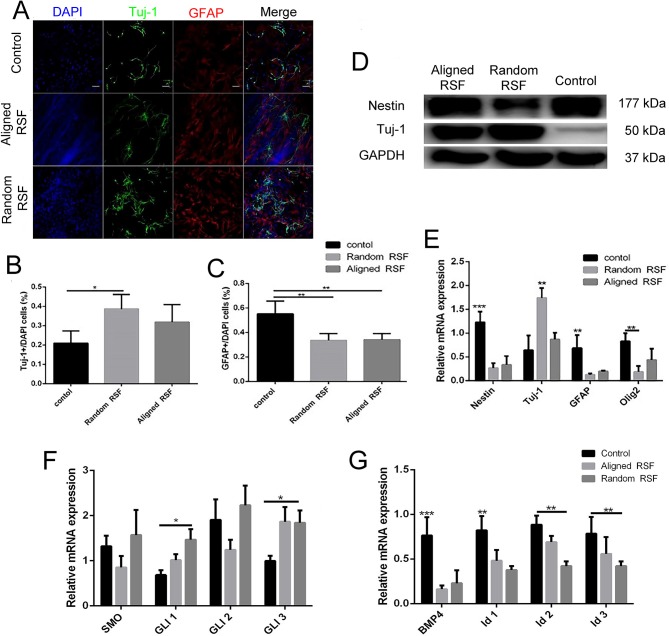
The differentiation of NPCs on coverslip controls and random and aligned RSF mats after culturing for 7 days. **(A)** Representative fluorescence images of differentiated NPCs under differentiation conditions. The cells were immunostained with Tuj-1 for neurons (green), GFAP for astrocytes (red), and DAPI for nuclei (blue). The percentages of Tuj-1+ cells **(B)** and GFAP+ cells **(C)** among differentiated NPCs in the three groups. **(D)** Western blot analysis of nestin and Tuj-1 protein expression of differentiated NPCs on coverslip controls and aligned and random RSF mats. **(E)** qPCR analysis of nestin, Tuj-1, GFAP, and Olig1 mRNA expression in differentiated NPCs cultured on the three substrates. **(F)** qPCR analysis of the relative mRNA expression of Smo and Gli1–3, which are involved in the SHH signal pathway, after 7 days of differentiation on the three substrates. **(G)** qPCR analysis of the relative mRNA expression of BMP4 and Id1–3, which are involved in the BMP4 signaling pathway, after 7 days of differentiation on the three substrates. The data are presented as the mean ± standard error of the mean, ^*^p < 0.05, ^**^p < 0.01, ^***^p < 0.001. Scale bar: 50 μm.

To investigate the phenotypic changes of differentiated NPCs on RSF mats and coverslips, NPCs were observed after culturing for 7 days in differentiation medium. After 7 days of differentiation, the cells exhibited elongated cell shapes with healthy neurite outgrowth, leading to a confluent neural network covering almost the whole RSF surface (Figures 3A,B). After 7 days differentiation, we used Tuj-1 as a neuron marker and GFAP as an astrocyte marker (Figure 6A). The differentiated cells were 18.89 ± 0.53%, 36.18 ± 0.61%, and 31.39 ± 1.31% Tuj-1 positive on control coverslips, aligned, and random RSF mats, respectively (Figure 6B), and were 54.20 ± 1.82%, 23.58 ± 0.96%, and 22.54 ± 0.94% GFAP positive on coverslips and aligned and random RSF mats, respectively (Figure 6C). There was a significant difference in the number of GFAP-positive cells on the aligned and random RSF mats compared to coverslips and a significant difference in the number of Tuj-1–positive cells on the random RSF mats compared to coverslips. Thus, the NPCs retained their ability to differentiate into different neural subtypes on the RSF mats. For a quantitative analysis, the total protein was extracted and subjected to western blot assay, and total RNA was extracted and subjected to real-time PCR and qPCR analysis after 7 days of culture in differentiation medium. The western blot analysis showed that compared with controls, NPCs cultured on aligned and random RSF mats exhibited significantly lower nestin expression, while the expression of Tuj-1 was enhanced in cells grown on aligned and random RSF mats (Figure 6D). The nestin mRNA expression was lower in cells grown on RSF mats compared to those grown on coverslips, while the Tuj-1 mRNA expression was significantly greater in cells grown on the RSF mats compared to those grown on coverslips (Figure 6E). Thus, both the aligned and random RSF mats greatly enhanced NPC differentiation into neurons, while the laminin-coating coverslips greatly enhanced NPC differentiation into astrocytes. We further analyzed the signaling factors that might be involved in this process of differentiation using real-time PCR and qPCR analysis after 7 days of culture. There was no significant difference in the SHH signaling pathway among the three groups (Figure 6F), while the expression of factors in the BMP4 signaling pathway was reduced in both the aligned and random RSF mats compared to coverslips (Figure 6G).

## Discussion

The use of NPCs holds great promise for the improvement of drug screening as well as for cell therapy-based treatment of neurological disorders such as Parkinson's disease (Ambasudhan et al., 2014; Le Grand et al., 2015), Huntington's disease, amyotrophic lateral sclerosis, traumatic spinal cord injuries, and peripheral nerve injuries (Kim et al., 2013; Ferrari et al., 2018). Previous studies in neural progenitor cell culture have relied primarily on animal or human-derived matrices (Teixeira et al., 2007). However, there are several issues with these matrices. (i) They cannot be transplanted together with the cells to the location of the injured tissue because they are usually not mechanically strong enough. (ii) Because of regulatory issues, potential clinical applications would require the cells to be grown on specific non-animal-derived matrices. (iii) The nature of undefined matrices might differ depending on the processing methods, making it difficult to draw conclusions from the experimental works. (iv) In order to mimic the natural ECM, the matrices need to be three dimensional to provide the mechanical framework to permit cell-cell interactions for healthy tissue formation and maintenance. In this study, we cultured NPCs using aligned and random RSF mats that can mimic the ECM of the cell, and these RSF mats can be transplanted together with cells into the injured site (Xu et al., 2017).

Cell adhesion, biocompatibility, and other biological effects of biomaterials are important for their tissue-engineering applications and for the repair of defective tissues, and interactions between cells and the ECM play a pivotal role in regulating biological tissue functions. NPCs can proliferate or differentiate into various cell types depending on the presence of suitable scaffolds and appropriate culture conditions. Thus, for the sake of spreading, proliferating, differentiating, and maintaining cellular functions, these cells require good attachment to a substrate. In the present study, good NPC adhesion was observed for both aligned and random RSF mats (Figures 3A,B), which is an important prerequisite for biological applications of RSF mats. The good adhesion of the NPCs to the RSF mats was mainly due to the unique surface properties of the RSF material itself and the laminin coating. SF is a protein consisting of up to 90% glycine, alanine, and serine amino acids and can be fully degraded by naturally occurring proteolytic enzymes (Dal Pra et al., 2005). The SF can be absorbed with the organism and is non-cytotoxic. The presence of the Arg-Gly-Asp amino acid sequence might act as a biological recognition signal and promote cell adhesion. Furthermore, previous studies have shown that different amino acid sequences and modifications to the surface of RSF—such as amines, alcohols, carboxyl groups, and thiols—can change the adhesion of cells to RSF (Teuschl et al., 2014; Zhao et al., 2014). For example, aspartic acid and glutamic acid carboxylic acid groups can be modified with primary amine peptides, such as RGD sequences, to improve cell adhesion. Therefore, SF is more suitable than other forms of biomedical materials because the structure of silk proteins can significantly affect cell adhesion to the surface (Zhang et al., 2005).

The biocompatibility of RSF mats can differ owing to differences in RSF synthesis techniques and different biological applications. In our experiment, the RSF mats were post-treated at high humidity, which makes RSF more biocompatible compared to RSF post-treated with 90 vol% aqueous ethanol solution during the drying period (Fan et al., 2013). Here, we used 75 vol% ethanol to sterilize the RSF mats for 2 h before seeding cells, then we used the sterilized PBS and culture medium to wash the mats in order to reduce the impact of the ethanol on the biocompatibility of the RSF. In this study, we found no obvious cytotoxicity for the RSF mats (Figure 4). The good biocompatibility of the RSF mats is consistent with previous studies showing that RSF matrices can used for urethral reconstruction (Xie et al., 2014) and that RSF is suitable for neurite outgrowth (Zhang et al., 2005). There might be two reasons for the good interactions between RSF mats and NPCs. First of all, SF is derived from nature, not artificially synthesized, and the electrospinning technique can produce RSF mats with high porosity. Sericin is a glue-like protein that holds the fibroin fibers together and has been identified as a source of immunogenic reactions (Panilaitis et al., 2003). Removal of sericin from the RSF mats reduces immnuoreactions and improves biological safety. Furthermore, the coating with laminin (a widely used biomolecule for NPC culture) is a standard protocol for the surface treatment of NPC-related biomaterial, and this might also produce a favorable surface on the RSF mats for NPCs. Collagen coating has recently been reported to offer a more cytocompatible RSF surface for adhesion, spreading, and growth of human corneal endothelial cells (Madden et al., 2011).

Most tissue engineering is based on a combination of biomaterials and stem/progenitor cells. The reason for this is that stem cells have the potential to differentiate into the desired cell types and to produce new ECM at the injured site. Our study demonstrates that RSF mats are a promising scaffold material for the growth of NPCs. Apart from the excellent biocompatibility, the RSF mats (both aligned and random) can stimulate NPC proliferation through the upregulation of Ki-67 expression (Figure 5H). They can also enhance NPC differentiation into astrocytes, especially neurons (Figure 6B), thus helping overcome the non-renewable nature of neuronal cells and their lack of differentiation. We found that the RSF mats stimulated the NPCs to differentiate into neurons by repressing BMP4 signaling factors, and this is consistent with a previous study (Gámez et al., 2013; Han et al., 2017). The BMP4 signal could induce NPC differentiation into astrocytes on laminin-coated coverslips, while the RSF mats did not stimulate differentiation through SHH signaling.

Once the cells were seeded on the RSF mats, they were capable of attaching, growing, and differentiating, which is consistent with earlier studies showing that substrates made from SF fibers support the survival and growth of attached neurons and dorsal root ganglion cells (Gennari et al., 2018), suggesting a potential use of RSF for preparing tissue-engineered nerve guides (Zhang et al., 2005; Sun et al., 2017) or drug delivery vehicles to treat CNS injuries or diseases. There are several factors that determine the regenerative capability of a scaffold, including scaffold porosity, pore size, and morphology; biodegradation profile; and biocompatibility (Wang et al., 2012). The aligned and random RSF mats have an appropriate structure to allow attached NPCs to proliferate and to differentiate into neurons. However, the detailed mechanisms of this process remain unknown. Further research is needed to investigate other biomaterials in combination with RSF and to explore the mechanism through which RSF mats influence NPC growth, proliferation, and differentiation.

## Conclusion

We have demonstrated the first use of RSF mats as robust scaffolds for NPC culture *in vitro*. The RSF mats can support NPC growth and keep the cells in a more active proliferation state than laminin-coated coverslips, and both aligned and random RSF mats enhance the differentiation of NPCs into neurons. Our findings thus indicate the great potential of RSF mats in NPC research, neural tissue engineering, and neural prostheses.

## Data Availability

All datasets generated for this study are included in the manuscript and/or the Supplementary Files.

## Author Contributions

SS, HL, and RC designed the biological experiments. YZ designed the RSF biomaterials. GL, KC, DY, MX, and WL performed the experiments. GL, SS, SF, YZ, and RC analyzed the data. GL and SS wrote the manuscript. All the authors have approved the final version of the manuscript.

### Conflict of Interest Statement

The authors declare that the research was conducted in the absence of any commercial or financial relationships that could be construed as a potential conflict of interest.

## Supplementary Material

Figure S1 SEM images of NPCs cultured on random and aligned RSF mats without laminin coating in proliferation medium after 3 days culture. Scale bar: 50 μm.
