# Supplementary figures and images for: Laminin-Coated Electrospun Regenerated Silk Fibroin Mats Promote Neural Progenitor Cell Proliferation, Differentiation, and Survival in vitro
Source: Front Bioeng Biotechnol. 2019 Aug 6;7:190. doi: 10.3389/fbioe.2019.00190 (PMC6691020; doi:10.3389/fbioe.2019.00190)

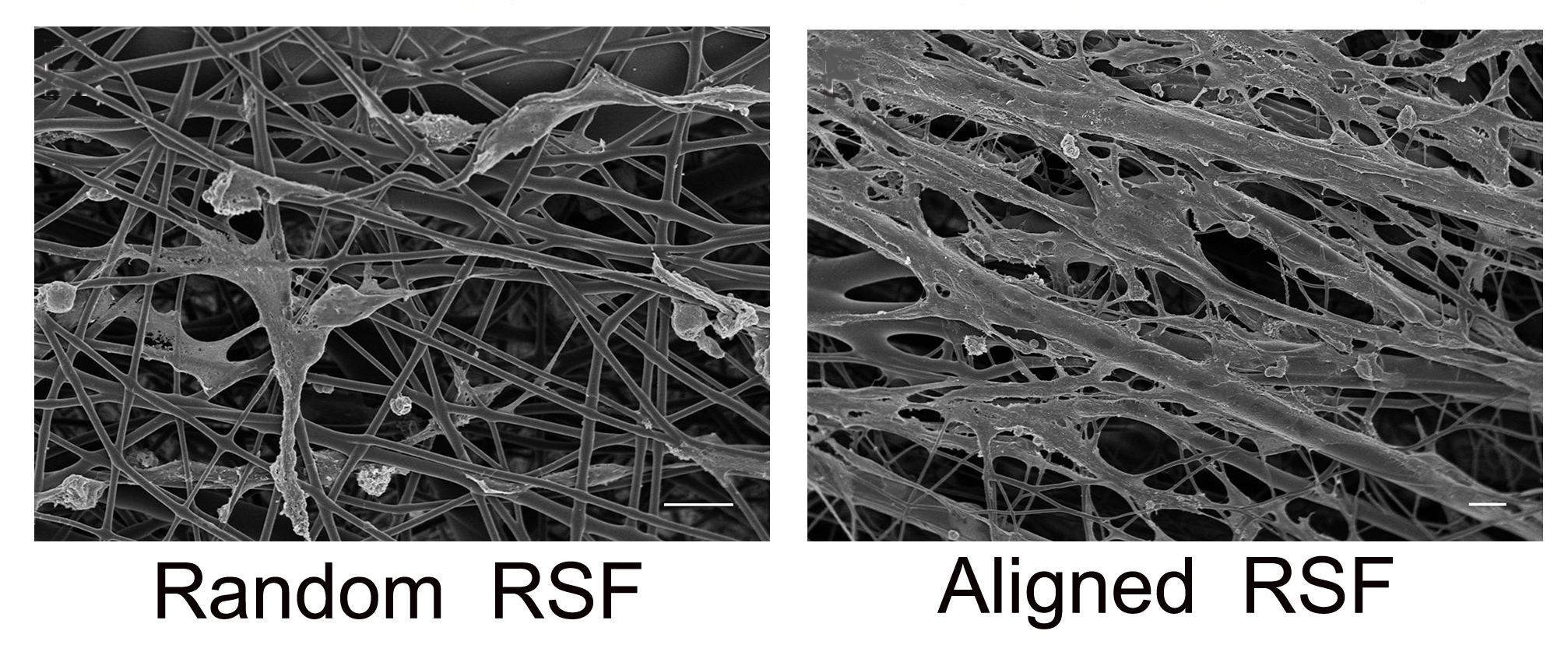

Supplement: Figure S1 — SEM images of NPCs cultured on random and aligned RSF mats without laminin coating in proliferation medium after 3 days culture. Scale bar: 50 μm. [file Image_1.JPEG]
